# Pediatric Congenital Tracheobronchial Variants and Their Clinical Significance for Endotracheal Intubation and Airway Management: A Scoping Review of Fetal, Neonatal, and Pediatric Cases

**DOI:** 10.7759/cureus.108003

**Published:** 2026-04-29

**Authors:** Spyridon Kanakaris, Theano Demesticha, Dimitrios Filippou, George Triantafyllou, George Tsakotos, Nikolaos Lazaridis, Maria Piagkou

**Affiliations:** 1 Department of Anatomy, School of Medicine, Faculty of Health Sciences, National and Kapodistrian University of Athens, Athens, GRC; 2 Department of Anatomy and Surgical Anatomy, School of Medicine, Aristotle University of Thessaloniki, Thessaloniki, GRC

**Keywords:** bridge bronchus, bronchial atresia, bronchial isomerism, bronchoscopy, endotracheal intubation, pediatric airway, pulmonary artery sling, scoping review, tracheal agenesis, tracheal bronchus

## Abstract

Congenital tracheobronchial variants (CTBVs) may distort airway landmarks, alter carinal recognition, and complicate ventilation during pediatric endotracheal intubation. Although uncommon, these anomalies can create important peri-intubation risks that are often underrecognized.

This scoping review was conducted in accordance with the Joanna Briggs Institute methodology and Preferred Reporting Items for Systematic Reviews and Meta-Analyses extension for Scoping Reviews (PRISMA-ScR) guidance. Using a Population-Concept-Context framework, we identified human case reports and small case series describing congenital tracheal or bronchial anomalies relevant to intubation, bronchoscopy, ventilation, or airway planning. Searches were performed in PubMed, Scopus, and Web of Science, with additional studies identified through manual searching.

Sixty-six records were identified. After removal of one duplicate, 61 records from the database search were screened, 42 were excluded, and 23 full-text reports were assessed. Twenty studies were included, comprising 18 case reports and two small case series. Six studies described tracheal agenesis or atresia, four bronchial atresia, three tracheal bronchi, three bridge bronchi with or without pulmonary artery sling, and four bronchial isomerism or laterality defects. Cases ranged from 21 weeks of gestation to 15 years of age. Tracheal agenesis or atresia produced the most severe cannot-intubate presentations. Fixed stenotic lesions increased risks during tube passage and sizing, whereas distal anomalies more commonly mimicked selective intubation, mucus plugging, or refractory wheeze.

CTBVs have important implications for pediatric airway management. Pre-intubation anatomical assessment and early bronchoscopic confirmation may reduce avoidable complications and support safer individualized airway planning.

## Introduction and background

Pediatric endotracheal intubation relies on predictable anatomical landmarks, including tracheal length, the level of bifurcation, right-left bronchial asymmetry, typical lobar branching, and the relationship of the airway to the great vessels. These landmarks help clinicians confirm tube position, recognize endobronchial misplacement, and interpret ventilation findings during airway management. However, congenital tracheobronchial variants (CTBVs) may disrupt these expected patterns and complicate both intubation and subsequent ventilation.

At the severe end of the spectrum, tracheal agenesis (TAg) prevents formation of a normal central airway and may allow only temporary ventilation through the esophagus [1-5]. In contrast, more distal abnormalities such as segmental bronchial atresia (BAt) may not prevent initial tracheal access but can present with unilateral hyperinflation, atelectasis, or unexplained abnormal breath sounds during positive-pressure ventilation [6-8]. These conditions may therefore appear either as a difficult airway or as unexplained ventilation failure after apparently successful intubation.

Several clinically important anomalies lie between these extremes. A tracheal bronchus (TB) may be unintentionally occluded by the endotracheal tube (ETT), causing right upper lobe (RUL) collapse or selective lobar ventilation. A bridge bronchus (BB) with a pseudocarina may mislead recognition of the true bifurcation and appropriate tube depth. A left pulmonary artery sling (LPAS) can compress the distal trachea, causing fixed narrowing. Bronchial isomerism (BI) may obscure normal right-left anatomy and distort bronchoscopic landmarks [9-17]. Recognition of these variants is important because they may influence tube selection, insertion depth, ventilation strategy, bronchoscopy, and perioperative planning.

Although clinically relevant, these anomalies are usually reported as isolated case reports across several specialties, including thoracic surgery, anesthesiology, radiology, neonatology, pulmonology, and pediatric cardiology. As a result, the available evidence remains fragmented and difficult to translate into a practical framework for clinicians, trainees, and multidisciplinary airway teams.

The aim of this scoping review was to synthesize reported fetal, neonatal, and pediatric cases of CTBVs with emphasis on anatomical clarity and clinical utility. The review examined both difficult airway events and anomalies affecting ventilation, landmark recognition, tube placement, and surgical planning. By organizing these reports into a structured framework, this review seeks to support safer pediatric intubation, improved airway management, and earlier recognition of uncommon congenital airway anatomy.

## Review

Methods

Study Design

This study was conducted as a retrospective scoping review in accordance with the Joanna Briggs Institute (JBI) methodology and the Preferred Reporting Items for Systematic Reviews and Meta-Analyses extension for Scoping Reviews (PRISMA-ScR) guidelines [18-21]. As the review was reconstructed from preserved records, no prospectively registered protocol was available. The Population-Concept-Context framework guided study eligibility, data extraction, and synthesis.

Eligibility Criteria

Eligible studies included fetal, neonatal, pediatric, or adolescent human subjects with congenital tracheal or bronchial abnormalities relevant to airway management, intubation, bronchoscopy, ventilation, or perioperative planning. Only peer-reviewed full-text reports containing patient-level data and sufficient anatomical detail were included. Secondary reviews, conference abstracts without full text, duplicate reports, and studies lacking adequate anatomical information were excluded. Small case series were retained when patient-level anatomical and airway-related data were available.

Search Strategy

Studies were identified through PubMed, Scopus, and Web of Science, supplemented by manual searching and citation tracking. The retrospective search covered all indexed years available within the preserved review record. Exact platform-specific search dates were unavailable and are reported transparently. The search strategy combined terms related to congenital airway anomalies, including tracheal agenesis, tracheal atresia, bronchial atresia, tracheal bronchus, bridge bronchus, pulmonary artery sling, bronchial isomerism, and laterality defect, together with pediatric population terms and airway-related terms such as endotracheal intubation, bronchoscopy, and ventilation.

Study Selection

**Figure 1:**
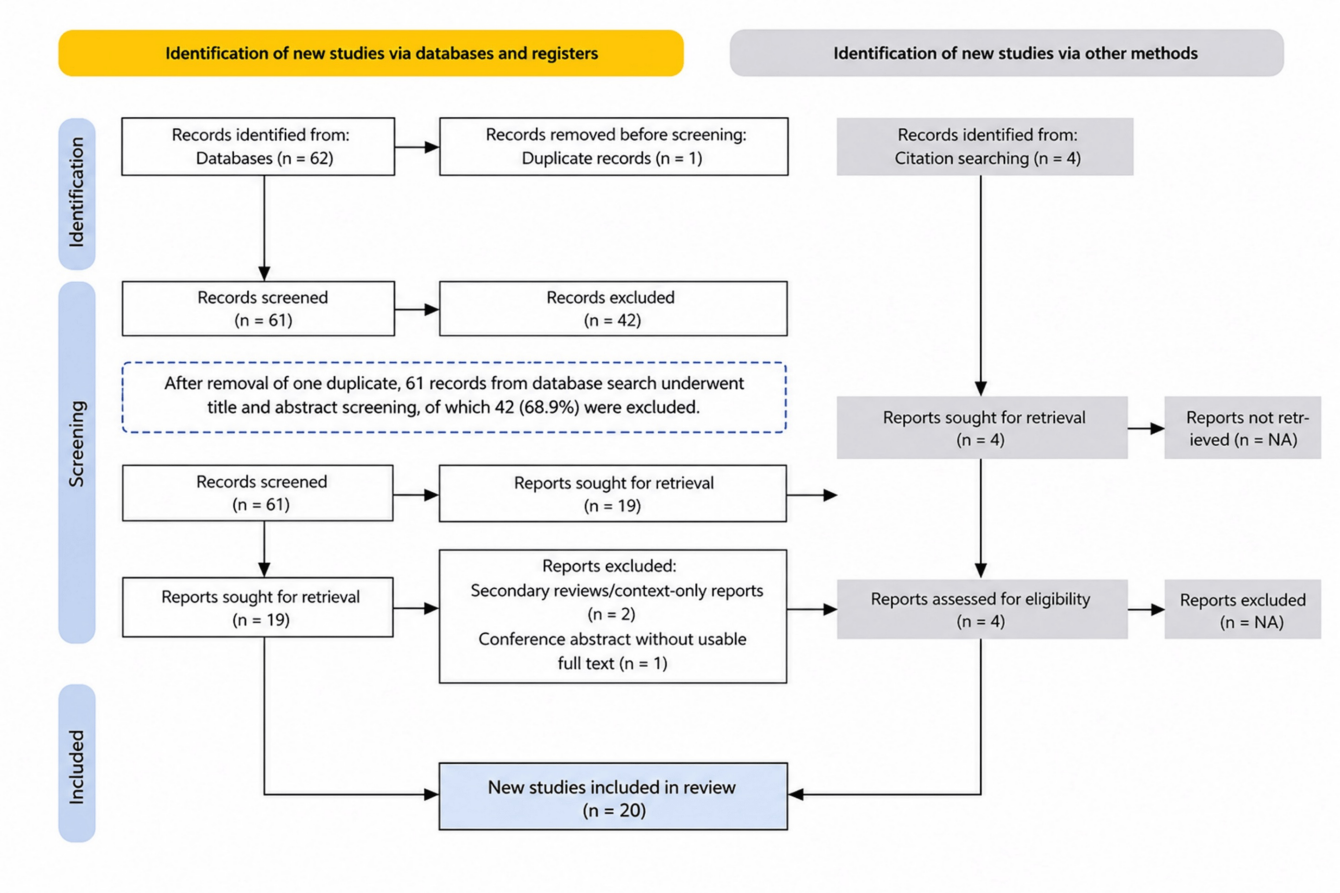
Flow diagram of study identification, screening, eligibility assessment, and inclusion. Records were identified through database searches (PubMed, Scopus, and Web of Science) and additional sources, including manual and citation searching. After duplicate removal, records underwent title and abstract screening, followed by full-text eligibility assessment. Studies meeting the predefined inclusion criteria were included in the final review corpus.

A total of 66 records were identified, including 62 (93.9%) from database searches and four (6.1%) from additional sources. After removal of one duplicate, 61 records from database search underwent title and abstract screening, of which 42 (68.9%) were excluded. Twenty-three full-text reports were assessed for eligibility. Three studies (13.0%) were excluded at this stage, leaving 20 included studies (87.0% of full-text reports) in the final review corpus. The study selection process is presented in Figure 1.

Data Extraction and Quality Assessment

Extracted variables included publication details, patient age, anatomical anomaly, laterality, clinical presentation, diagnostic findings, treatment, outcome, and relevance to intubation, bronchoscopy, ventilation, or perioperative airway management. Screening and charting were performed using predefined criteria and consolidated into a reconciled dataset. Reporting completeness was assessed using the CARE reporting domains for case reports and adapted JBI critical appraisal tools for case series [22-27]. These assessments were used to describe reporting quality rather than to exclude studies.

Data Synthesis

Because of marked heterogeneity in anatomical patterns, age range, clinical presentation, and reported outcomes, formal meta-analysis was not appropriate. Findings were therefore synthesized using descriptive counts and thematic analysis [28], with emphasis on pediatric endotracheal intubation, airway management, and interpretation of ventilation findings.

Results

Study Selection

The study selection process is summarized in Figure 1. A total of 66 records were identified, including 62 (93.9%) from PubMed, Scopus, and Web of Science, and 4 (6.1%) from manual searching and other sources. After removal of one duplicate, 65 records underwent title and abstract screening, of which 42 records (68.9%) were excluded. Twenty-three full-text reports were assessed for eligibility. Three reports (13.0%) were excluded at the full-text stage, comprising two background reviews and one conference abstract without sufficient chartable anatomical data. A final total of 20 studies (87.0% of full-text reports assessed) were included in the review corpus.

Overview of the Corpus

**Figure 2:**
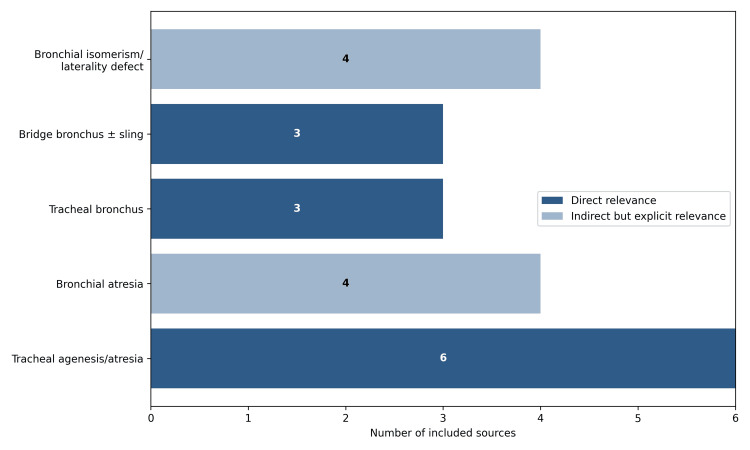
Distribution of included studies by anomaly class and airway relevance. This figure presents the number of studies included, categorized by congenital tracheobronchial anomaly (CTBA) class and stratified by level of airway relevance. Dark bars represent studies directly relevant to airway management, intubation, or bronchoscopy, whereas lighter bars indicate studies with indirect but explicit relevance through effects on ventilation interpretation, anatomical landmarks, or peri-intubation planning.

Figure 2 and Table 1 summarize the included studies. The final corpus comprised 20 studies published between 1974 and 2024, including 18 case reports (90.0%) and two small case series (10.0%). Twelve studies (60.0%) were directly focused on endotracheal intubation, bronchoscopy, or airway management. The remaining eight studies (40.0%) were indirectly relevant because they influenced the interpretation of unilateral aeration, bronchoscopic landmarks, or response to positive-pressure ventilation.

**Table 1:** Characteristics of the 20 included studies and dominant peri-intubation pitfalls. This table summarizes the included studies according to source type, clinical presentation window, index congenital anomaly, anatomical topography, level of airway relevance, and the principal peri-intubation pitfall identified in each report. Direct airway relevance refers to studies primarily focused on airway anatomy, endotracheal intubation, bronchoscopy, or perioperative airway management. Indirect but explicit relevance refers to studies not centered on intubation itself but with clear implications for ventilation, tube positioning, radiographic interpretation, or airway planning. BAt, bronchial atresia; BB, bridge bronchus; BI, bronchial isomerism; CR, case report; CS, case series; LUL, left upper lobe; LUS, lung ultrasound; NB, newborn; PAS, pulmonary artery sling; PEEP, positive end-expiratory pressure; PPV, positive-pressure ventilation; RUL, right upper lobe; TAg/TAt, tracheal agenesis/tracheal atresia; TB, tracheal bronchus; TEF, tracheoesophageal fistula; T4-T5/T6-T7, thoracic vertebral levels; LDs, laterality defects

Study	Source type	Clinical window	Index anomaly and topography	Airway relevance	Dominant peri-intubation pitfall
Lopez et al. [1]	CR	NB 37 weeks, 2.30 kg	TAg/TAt, subglottic cul-de-sac, no thoracic trachea	Direct	Cannot intubate with paradoxical esophageal ventilation
van der Putten et al. [2]	CR	NB 30+5 weeks, 1,750 g	TAg/TAt, 5-mm subglottic tract	Direct	Cannot intubate; avoid repeated attempts
Ali et al. [3]	CR	NB 30 weeks, 1,290 g	TAg/TAt, 6-cm blind-ending "trachea" + large TEF	Direct	Temporary transesophageal ventilatory bridge
Baroncini-Cornea et al. [4]	CR	NB 35 weeks, 2 kg	TAg/TAt, tracheal gap 52.9 → 67.9 mm	Direct	Subglottic stop 1.2 mm from the cords; transesophageal rescue
Bhattarai et al. [5]	CR	NB 28 weeks, 2.0 kg	TAg/TAt, no trachea below the thyroid cartilage	Direct	Unstable esophageal ventilation during manipulation
Williams et al. [6]	CR	Boy, 14 years	BAt, apical segment of the RUL, 3 cm distal	Indirect but explicit	Air trapping mimicking selective intubation
Haller et al. [7]	CR	Birth → 10 years	BAt, apicoposterior segment, LUL	Indirect but explicit	PEEP/PPV is liable to aggravate a tension effect
Rebollo-Simarro & Alonso-Ojembarrena [8]	CR	Term neonate	BAt, left upper lobe, exact level incompletely defined	Indirect but explicit	Deceptive distal hyperinflation; bedside LUS helpful
Sharma et al. [9]	CR	Girl, 2 years	TB, supracarinal origin of the right upper lobe bronchus	Direct	Occlusion of the aberrant bronchus by the tip/cuff
Lovett et al. [10]	CR	Girl, 2 years	TB, distal trachea ~2 mm + bronchus suis	Direct	Critical tube sizing + risk of bronchus suis obstruction
Pardo et al. [11]	CR	Boy, 11–14 years	BB ± PAS, pseudocarina T6–T7, BB arising from left main bronchus	Direct	Carina/pseudocarina confusion
Baden et al. [12]	CS	3 cases, 6 days → 6 years	BB ± PAS, true carina T4–T5, pseudocarina T6–T7	Direct	Endobronchial intubation from pseudocarina misidentification
Li et al. [13]	CR	Infant, 10 months	BB ± PAS, BB + PAS + partial airway narrowing	Direct	Inadvertent selectivity + need for bronchoscopic confirmation
Cappuccio et al. [14]	CR	Infant, 1 month	BI/LD, bilateral hyparterial bronchi, bilateral bilobed lungs	Indirect but explicit	Altered bronchoscopic lateral landmarks
Adhikari et al. [15]	CR	Girl, 4 years	BI/LD, bilateral hyparterial bronchi	Indirect but explicit	Landmark error for selective intubation/blocker
Lee et al. [16]	CR	Boy, 12 years	BI/LD, left isomerism + bilateral dynamic stenoses	Indirect but explicit	Bronchomalacia mimicking bronchospasm or mainstem intubation
Bush [17]	CS	3 cases, 4–15 years	BI/LD, long main bronchi + diffuse malacia	Indirect but explicit	Tube depth difficult to judge; collapse at extubation
Martínez et al. [29]	CR	Fetus → NB 38 weeks	BA, right main bronchus; cul-de-sac ~2 cm from the bifurcation	Indirect but explicit	Mediastinal shift may simulate malposition
Ashmeade & Carver [30]	CR	Term neonate	TB, right TB, distance not reported	Direct	Iatrogenic RUL atelectasis related to tube position
Krishnamurthy et al. [31]	CR	NB 32+1 weeks	TAg/TAt, 3-cm proximal trachea, disconnected distal segment	Direct	Neither the tracheal nor the esophageal route is functional

By anomaly type, six studies (30.0%) described TAg or TAt, four (20.0%) BAt, three (15.0%) TB, three (15.0%) BB with or without PAS, and four (20.0%) BI or laterality defects (LDs). This distribution indicates that the available evidence was not dominated by a single lesion type but rather represented several distinct congenital airway variants with different implications for pediatric airway management.

Age Range and Clinical Presentation

The included cases ranged from 21 weeks of gestation to 15 years of age, demonstrating that congenital tracheobronchial anomalies may present across the full pediatric age spectrum. Severe central airway abnormalities, such as TAg or TAt, were most commonly identified during the fetal or neonatal period because of immediate respiratory compromise. In contrast, more distal lesions such as BAt or TB were often diagnosed later in infancy or childhood after recurrent respiratory symptoms, abnormal imaging, or difficulties during intubation.

Airway Management and Ventilation Findings

Several anomalies had direct implications for airway management. TB increased the risk of ETT obstruction, potentially resulting in RUL collapse. BB with a pseudocarina could mislead recognition of the true bifurcation and contribute to incorrect tube placement. PAS and complete tracheal rings were associated with fixed airway narrowing, increasing the risk of tube mismatch, traumatic intubation, or resistance during tube advancement. Other anomalies primarily affected the interpretation of ventilation rather than tube placement itself. BAt and LDs frequently mimicked selective ETT, mucus plugging, bronchospasm, or unilateral hyperinflation. Failure to consider congenital anatomical variation could therefore delay recognition of the underlying anomaly.

Thematic Synthesis

Five major themes emerged from the included literature: anatomical topography, age-related clinical phenotype, airway mapping, mechanisms of intubation difficulty or ventilatory misinterpretation, and treatment strategies with peri-intubation implications. Overall, the findings support careful pre-intubation anatomical assessment and a low threshold for bronchoscopic confirmation in children with known or suspected TBVs.

Age Range, Diagnostic Methods, and Clinical Relevance

The developmental spectrum of the included cases was broad, ranging from fetal detection at 21 weeks of gestation to reassessment in adolescence, with reported cases at ages 12, 14, and 15. Despite this wide age range, neonatal and preterm presentations predominated, reflecting the greater clinical impact of central airway abnormalities that commonly become apparent soon after birth.

The most frequently reported diagnostic modalities were bronchoscopy or endoscopy, used in 14 of 20 studies (70.0%), followed by computed tomography (CT)-based imaging, including CT, CTA, or multidetector CT (MDCT), reported in 13 studies (65.0%). Conventional contrast studies or bronchography were described in six studies (30.0%), while surgical or autopsy anatomical confirmation was also reported in six studies (30.0%). Functional physiological assessment was noted in five studies (25.0%), and targeted prenatal or fetal US in three studies (15.0%).

As shown in Table 1, the distribution of airway relevance was markedly polarized. All studies describing TAg or TAt, BB, and TB had direct implications for airway management, endotracheal intubation, or bronchoscopy. In contrast, studies describing distal BAt and LDs were included because of their indirect but clinically important relevance, particularly their potential to mimic selective intubation, unilateral obstruction, persistent atelectasis, or otherwise unexplained ventilation abnormalities.

Anatomical Typology and Topography of Variation

The first theme examined the anatomical level and spatial distribution of the reported variants. The included studies demonstrated a broad continuum, ranging from peripheral segmental lesions, in which tracheal access may remain intact, to central defects in which a continuous airway was absent. This distinction was clinically important because distal abnormalities were more likely to present as ventilation or imaging abnormalities, whereas central lesions more often caused immediate airway compromise.

At the distal end of this spectrum, Williams et al. [6] described atresia of the apical segmental bronchus of the RUL, located approximately 3 cm from its origin. Haller et al. [7] reported segmental atresia involving the apicoposterior region of the left upper lobe (LUL). Rebollo-Simarro and Alonso-Ojembarrena [8] described atresia affecting LUL with associated subsegmental paramediastinal atelectasis. Although initial tracheal intubation may be uncomplicated in such cases, unilateral hyperinflation, collapse, or persistent asymmetrical ventilation may be misinterpreted during positive-pressure ventilation.

The BB represented a different anatomical pattern, characterized not by interruption but by abnormal redistribution of bronchial branching. Pardo et al. [11] described a BB with a pseudocarina at the T6-T7 level and origin of the intermediate bronchus from the left main bronchus, together with a LPAS passing between the trachea and esophagus. Baden et al. [12], in a series of three children, identified a true carina at the T4-T5 level and a more distal pseudocarina at the T6-T7 level. In these patients, the RUL was supplied by the right main bronchus, whereas the right middle and lower lobes received contralateral supply through the BB. Li et al. [13] also reported a BB associated with PAS and partial airway narrowing. These variants may mislead clinicians during bronchoscopy or tube positioning if the pseudocarina is mistaken for the true bifurcation.

The TB formed a third anatomical group characterized by supracarinal branching. Sharma et al. [9] described a right TB supplying the RUL, associated with mild distal tracheal stenosis (TS). Ashmeade and Carver [30] reported a similar right TB in a newborn with congenital diaphragmatic hernia. Lovett et al. [10] described a more complex variant consisting of a right bronchial sinus, severe distal TS of approximately 2 mm, complete tracheal rings, and PAS. In these situations, an ETT may inadvertently obstruct the aberrant bronchial origin, resulting in RUL collapse or unexplained desaturation.

Central TAg or TAt represented the most severe morphological group in the corpus. Lopez et al. [1] described a complete TAg (Floyd II type) with an immediate subglottic blind pouch, absence of the thoracic trachea, and a short communication between the bronchi and esophagus. van der Putten et al. [2] reported a Floyd III/Faro B variant with only a short proximal segment followed by a complete absence of the remaining trachea. Other reports described discontinuous proximal and distal tracheal segments, blind-ending tracheal structures, bronchoesophageal carinae, or progressive widening of long tracheal gaps [3-5,31]. These anomalies were associated with the most severe cannot-intubate presentations and often required emergency rescue ventilation or complex surgical planning.

LDs formed a separate topographical category in which the abnormality reflected altered symmetry rather than interruption or ectopic branching. Cappuccio et al. [14] described bilateral bilobed lungs, bilateral hyparterial bronchi, and elongated main bronchi consistent with left BI. Similar findings were reported by Adhikari et al. [15]. Lee et al. [16] linked left isomerism with multifocal bronchomalacia and dynamic narrowing of both main bronchi. Bush [17], in a series of three children, confirmed bilateral left-sided bronchial morphology with diffuse bronchomalacia. These patterns may complicate recognition of normal right-left bronchial landmarks and affect interpretation of selective ventilation or bronchoscopy findings.

**Figure 3:**
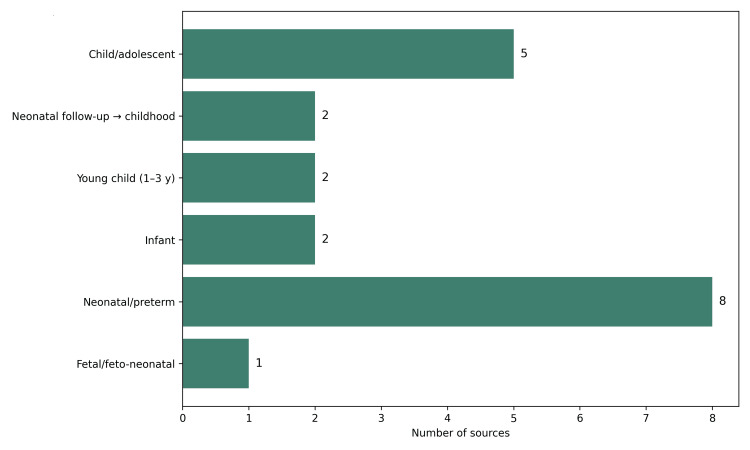
Distribution of sources by clinical age group. This figure presents the number of included studies according to clinical age group. The largest proportion of reports involved neonatal or preterm patients, followed by child or adolescent populations. Smaller numbers of studies were identified in fetal or feto-neonatal, infant, and early childhood age groups. This distribution suggests that severe central airway anomalies are more commonly recognized in the neonatal period, whereas less acute or diagnostically delayed conditions may present later in childhood.

Overall, the included studies illustrated a clear anatomical gradient, extending from peripheral segmental obstruction measured in distal centimeters to central airway absence measured in millimeters or long-gap defects. For airway management, this continuum had direct practical relevance. Depending on the lesion type, the main challenge may involve establishing an airway, identifying the true carina, avoiding occlusion of an aberrant bronchial origin, or interpreting asymmetric ventilation in a bronchial tree with non-standard laterality (Figure 3).

Clinical Phenotype and Age Window: From Fetal Hydrops to Refractory Asthma in Adolescents

The second theme concerned clinical presentation across age groups. The included studies covered a wide developmental spectrum, from the fetal period to late adolescence, and showed that similar anatomical variants could be presented in very different ways. Depending on lesion severity and location, presentation ranged from immediate respiratory collapse at birth to recurrent infection, infant stridor, feeding difficulty, or asthma-like symptoms later in childhood.

At the earliest end of the spectrum, Martínez et al. [29] described a lesion first detected at 21 weeks of gestation, with progression between 23 and 26 weeks to polyhydramnios, pleural effusion, skin edema, and thoracic compression. After 38 weeks of delivery, the newborn required prolonged non-invasive respiratory support until day 56 of life. These findings illustrate that severe congenital airway abnormalities may become clinically significant before birth and influence both fetal development and postnatal respiratory adaptation.

The most acute subgroup involved neonatal cannot-intubate scenarios, mainly associated with TAg or TAt. Lopez et al. [1] reported a term infant with cyanosis and severe distress in whom conventional intubation failed, but temporary improvement occurred after esophageal tube placement. van der Putten et al. [2] described a premature infant in whom the glottis could be visualized, yet the tube could not be advanced. Krishnamurthy et al. [31] reported a preterm neonate with gasping respirations, repeated airway failure, and rapid death. In this case, associated esophageal atresia (EAt) also prevented transesophageal rescue ventilation. Similar presentations of immediate distress and failed intubation were described by Ali et al. [3], Baroncini-Cornea et al. [4], and Bhattarai et al. [5]. These reports emphasize that severe central airway anomalies may present as unexpected failed-airway emergencies in the delivery room or neonatal intensive care unit.

Not all lesions were immediately catastrophic. Several cases first presented in infancy with unexplained respiratory symptoms or recurrent infection. Rebollo-Simarro and Alonso-Ojembarrena [8] described a term infant without overt distress in whom the anomaly was detected mainly through imaging. Cappuccio et al. [14] reported a one-month-old infant with growth restriction and recurrent respiratory infections. Li et al. [13] described a 10-month-old infant with stridor, dyspnea, and fever, consistent with airway compression and stenosis. Ashmeade and Carver [30] reported delayed diagnosis of TB after recurrent right upper lobe atelectasis, with bronchoscopy performed on day 49 of life. These cases show that congenital variants may initially mimic common neonatal or infant respiratory disorders.

Other patients presented later in childhood with chronic or intermittent symptoms. Sharma et al. [9] described a two-year-old child with recurrent chest infections from birth. Lovett et al. [10] reported a two-year-old with noisy breathing, poor weight gain, dysphagia, and later respiratory distress. Adhikari et al. [15] described a four-year-old with recurrent infections despite preserved oxygen saturation and no major hemodynamic instability. Pardo et al. [11] reported an older child with recurrent infections, desaturation episodes, and a high-risk asthma phenotype. Lee et al. [16] described a 12-year-old boy whose symptoms had long been treated as corticosteroid-resistant asthma before the underlying anomaly was recognized.

Peripheral BAt and LDs were often compatible with prolonged survival and were sometimes detected incidentally. Williams et al. [6] described a child who remained asymptomatic until adolescence despite abnormal imaging findings earlier in life. Haller et al. [7] reported progression from neonatal radiological findings to exertional dyspnea at 10 years of age. Baden et al. [12] and Bush [17] also demonstrated marked variation in the timing of diagnosis, ranging from the neonatal period to mid-adolescence.

Overall, three broad clinical windows emerged. The first was the neonatal period, dominated by failed-airway and cannot-intubate presentations in severe central anomalies [1-5,31]. The second involved infancy and early childhood, characterized by stridor, feeding difficulty, lobar collapse, or recurrent infection [8-10,13,14,30]. The third occurred in later childhood or adolescence, when anomalies were more likely to appear as refractory asthma, exercise intolerance, or chronic asymmetric ventilation [6,7,11,16,17]. These age-related patterns likely reflect both anatomical severity and the developing airway’s ability to compensate over time (Figure 3).

Diagnostic Mapping of the Airway: Methods, Measurements, and What They Capture or Miss

The third theme focused on diagnostic mapping of the airway. Beyond identifying which imaging or endoscopic tools were used, the included studies showed that each modality offered distinct strengths and limitations. Some techniques were useful for defining airway geometry and fixed stenosis, whereas others better demonstrated dynamic collapse, functional impairment, or relationships with adjacent vascular structures. In clinical practice, accurate diagnosis often depends on combining complementary modalities rather than relying on a single investigation.

In distal BAt, previous reports highlighted the value of multimodal assessment. Williams et al. [6] localized the atretic segment approximately 3 cm distally using bronchography and demonstrated absent ventilation and perfusion to much of the RUL on scintigraphy. Haller et al. [7] further quantified trapped gas using pulmonary function testing. Total lung capacity measured by helium dilution was 86.5% of predicted, whereas body plethysmography estimated total intrathoracic gas volume at approximately 152% of predicted, indicating a poorly ventilated compartment inaccessible to gas dilution. Xenon washout also showed prolonged expiratory clearance. These findings help explain why positive-pressure ventilation may worsen hyperinflation rather than improve gas exchange.

CT techniques, including CT, CTA, and MDCT, were widely used throughout the corpus. Pardo et al. [11] employed angiographic MDCT with three-dimensional reconstruction and minimum-intensity projection imaging to distinguish the true carina from a pseudocarina at the T6-T7 level and to demonstrate a left PAS between the trachea and esophagus. Li et al. [13] used volumetric imaging to identify abnormal origin of the left pulmonary artery, tracheal compression, BB, and right lower lobe atelectasis. Repeat imaging six months later demonstrated improvement in bronchial narrowing after vascular decompression. Lovett et al. [10] demonstrated the morphometric value of CTA by measuring the distal trachea at approximately 2 mm and correlating these findings with bronchoscopy. Sharma et al. [9] visualized a right TB and mild distal stenosis using multiplanar CT reconstruction, while Ashmeade and Carver [30] confirmed a similar lesion with bronchoscopy and CTA. These reports indicate that CT-based imaging was particularly valuable for airway dimensions, lesion level, and vascular relationships relevant to tube selection and procedural planning.

In central anomalies such as TAg or TAt, imaging and endoscopy were essential for determining whether any usable airway existed. Lopez et al. [1] combined tracheography and esophagography to demonstrate a subglottic blind pouch with contrast reaching the bronchi through the esophagus. van der Putten et al. [2] used tracheoscopy and CT to identify a short proximal airway segment measuring approximately 5 mm. Krishnamurthy et al. [31] highlighted radiographic warning signs during resuscitation, including a coiled nasogastric tube and an absent gastric bubble, before autopsy confirmed multiple discontinuous airway segments. Ali et al. [3] used microlaryngobronchoscopy to identify a blind-ending 6 cm tracheal structure with a large tracheoesophageal fistula. Baroncini-Cornea et al. [4] employed three-dimensional CT to measure a tracheal gap of 52.9 mm at birth, increasing to 67.9 mm at six months, followed by transesophageal endoscopy to map the distal carina and bronchi. Bhattarai et al. [5] also provided functional bedside evidence, showing improved oxygen saturation after distal esophageal ligation, confirming that the bronchi were being ventilated through the esophagus.

Ultrasound (US) and fetal imaging served a different but important role. Rebollo-Simarro and Alonso-Ojembarrena [8] used serial lung US to describe persistent focal consolidation and stable topographic abnormalities before CT, which were later confirmed as hyperinflation, relative hypovascularity, and air trapping. Martínez et al. [29] combined fetal US, fetal MRI, and fetal tracheobronchial endoscopy to guide prenatal intervention. In that case, the observed-to-expected lung-to-head ratio improved from 18% before decompression to 85% at 35 weeks, with amnioreduction of 1,400 mL. Adhikari et al. [15] used contrast-enhanced thoracoabdominal CT mainly to characterize bronchial and visceral laterality rather than airway stenosis.

LDs and bronchomalacia also demonstrated that static imaging alone may be insufficient. Lee et al. [16] combined CT, barium swallow, bronchoscopy, bronchography, and respiratory physiology, showing severe airflow obstruction not explained by asthma alone. Bush [17] similarly reported markedly abnormal lung function in children with bronchial isomerism and diffuse bronchomalacia. Cappuccio et al. [14] used contrast-enhanced CT to define bilateral hyparterial bronchi, elongated main bronchi, bronchiectasis, and airway wall thickening, with additional molecular confirmation of Kabuki syndrome.

The relative strengths of different modalities were most clearly summarized by Baden et al. [12]. In their series, bronchoscopy accurately assessed stenosis severity and airway length but could confuse the true carina with a pseudocarina. MDCT with virtual bronchoscopy was superior for defining branching origin and aerovascular anatomy, whereas complete tracheal rings remained primarily an endoscopic diagnosis.

**Figure 4:**
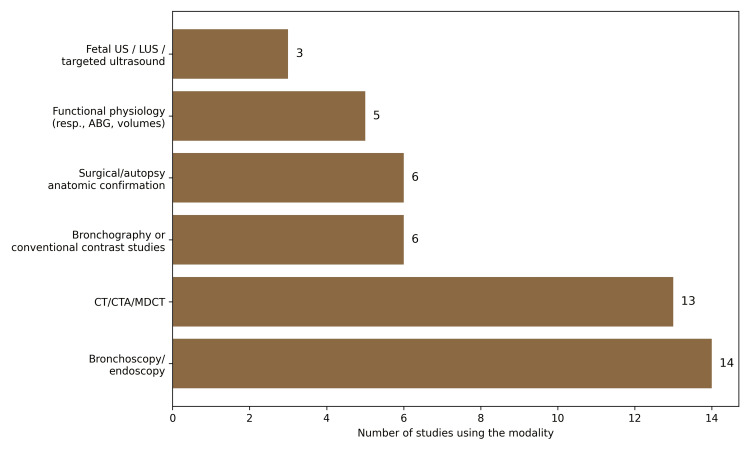
Frequency of diagnostic modalities used across the corpus. This figure illustrates the frequency of diagnostic modalities reported across the included corpus of studies. Bronchoscopy or endoscopy and computed tomography–based imaging (CT, CTA, or MDCT) were the most commonly used diagnostic tools, reflecting their central role in defining airway anatomy, lesion level, stenosis, and branching abnormalities. Less frequently reported modalities included bronchography or conventional contrast studies, surgical or autopsy confirmation, functional physiological assessments, and fetal or targeted ultrasound techniques. The distribution highlights the dominant role of endoscopic and CT-based imaging in the diagnostic evaluation of congenital pediatric tracheobronchial anomalies. LUS, lung ultrasound; CTA, computed tomography angiography; MDCT, multidetector CT

Overall, three practical conclusions emerged. First, CT-based imaging provided essential geometric information for safe airway management, including airway diameter, lesion level, pseudocarina location, and vascular compression. Second, bronchoscopy remained indispensable for dynamic findings such as malacia and complete rings, and for confirming true luminal anatomy. Third, functional testing and bedside ventilation markers helped explain abnormal physiology during positive-pressure ventilation and reduced the risk of misinterpreting congenital variation as simple tube malposition. Figure 4 summarizes this diagnostic hierarchy and highlights the dominant roles of endoscopy and CT in pediatric airway mapping.

Clinical Significance for Endotracheal Intubation: Modes of Failure and Pitfalls in Interpreting Ventilation

The central theme of this review was how CTBVs translate into practical difficulties during ETT and ventilation. Across the included studies, these anomalies were associated with complete airway failure, resistance to tube passage, misinterpretation of airway landmarks, or misleading ventilatory findings despite apparently successful intubation.

TAg or TAt represented the most severe form of binary airway failure, in which conventional tracheal intubation was impossible. Lopez et al. [1] described the inability to advance the tube below the glottis, with transient improvement after placement of an esophageal tube. van der Putten et al. [2] reported a similar scenario and emphasized avoidance of repeated traumatic attempts once passage was no longer possible. Baroncini-Cornea et al. [4] described subglottic arrest only 1.2 mm below the vocal cords, requiring emergency transesophageal ventilation. Ali et al. [3] demonstrated that esophageal ventilation could temporarily sustain life for approximately 60 hours when a large tracheoesophageal fistula connected the esophagus to the bronchi. Bhattarai et al. [5] further illustrated the fragility of this rescue pathway, with oxygen saturation falling to 70% during esophageal traction. These reports indicate that some neonates with failed intubation may only be oxygenated through a fistulous connection rather than a true tracheal airway.

Krishnamurthy et al. [31] highlighted an important exception: not all cases of TAg have a functional esophageal rescue route. In the presence of proximal EAt and absent distal communication, neither tracheal nor esophageal ventilation was possible. In such situations, the combination of failed intubation, a coiled nasogastric tube, and an absent gastric bubble should raise suspicion of complete tracheoesophageal discontinuity. This distinction has immediate clinical importance because it separates anomalies that may be temporarily rescued by esophageal ventilation from those incompatible with emergency airway creation.

Fixed stenoses and aberrant supracarinal branches created a different set of hazards. Sharma et al. [9] described a right TB that is vulnerable to obstruction by the ETT tip or cuff, with a risk of RUL collapse. Lovett et al. [10] reported a more severe scenario involving a distal trachea measuring approximately 2 mm, complete tracheal rings, and bilateral bronchomalacia. In such patients, tube size, insertion depth, and fiberoptic guidance may be critical. Ashmeade and Carver [30] showed that in an infant with congenital diaphragmatic hernia and limited pulmonary reserve, occluding a TB resulted in persistent RUL atelectasis. PAS with associated tracheal narrowing further increased the risk of traumatic intubation or inability to pass an appropriately sized tube.

The BB primarily altered recognition of the carina rather than tracheal access itself. Pardo et al. [11] emphasized that a pseudocarina at the T6-T7 level could be mistaken for the normal bifurcation, leading to unintentional selective intubation. Baden et al. [12] showed that confusion between the true carina and pseudocarina could occur even during bronchoscopy and was associated with hypoventilation, atelectasis, and difficulty weaning from ventilation. Li et al. [13] confirmed that when a BB coexisted with PAS and airway narrowing, clinicians might encounter incorrect tube depth, preferential ventilation, or unexplained lobar hypoventilation. In this subgroup, intubation may appear technically successful, yet the internal landmarks no longer correspond to expected anatomy.

Distal BAt was among the most misleading lesion groups because it often created false impressions of selective intubation. Williams et al. [6] reported that collateral hyperinflation of an affected lobe could be mistaken for excessive tube advancement into a main bronchus. Haller et al. [7] demonstrated that positive-pressure ventilation, excessive positive end-expiratory pressure, or inadequate expiratory time could worsen this trapped-air effect and mimic mainstem intubation or mucus plugging. Rebollo-Simarro and Alonso-Ojembarrena [8] suggested bedside lung US as a useful method to distinguish distal congenital pathology from tube malposition. Martínez et al. [29] further showed that marked thoracic asymmetry with a mediastinal shift in fetal and neonatal life could be mistaken for tube-related pathology, even when the true cause was right main BAt with bilobar deformation.

LDs and diffuse bronchomalacia interfered with both landmark recognition and interpretation of ventilatory resistance. Cappuccio et al. [14] and Adhikari et al. [15] showed that bilateral hyparterial bronchi and bilateral bilobed lungs altered the usual logic of selective intubation or bronchial blocker placement. Lee et al. [16] demonstrated that associated bronchomalacia may mimic refractory bronchospasm or endobronchial intubation because of dynamic collapse of the main bronchi. Bush [17] confirmed that elongated main bronchi and diffuse malacia reduced the reliability of standard depth estimates and increased the risk of airway collapse during induction or extubation. These reports illustrate that some high-risk congenital airway variants present not as failed intubation, but as misleading breath sounds, abnormal ventilator pressures, or confusing radiographic findings.

Overall, the included studies supported three broad categories of airway risk. The first involved central access failure, typical of TAg or TAt, in which successful rescue depended on the presence of a functional tracheoesophageal fistula. The second involved fixed narrowing, such as complete tracheal rings or vascular compression, where tube sizing, resistance to insertion, and mucosal trauma were major concerns. The third involved interpretative false positives, including TB, BB, distal BAt, LDs, and malacia, in which intubation may be technically successful but clinical interpretation can become dangerously misleading.

Therapeutic Strategy, Clinical Outcome, and Consequences for Peri-Intubation Planning

The fifth theme explored how anatomical variation influenced treatment strategy, clinical outcome, and peri-intubation planning. Management ranged from definitive surgical correction to supportive care, staged reconstruction, or conservative follow-up. Practical implications extended beyond surgery itself and included airway planning, tube selection, postoperative ventilation, and long-term respiratory monitoring.

In peripheral BAt, treatment most commonly involved surgical resection, with generally favorable outcomes. Williams et al. [6] reported right upper lobectomy followed by discharge on postoperative day eight and complete return to normal activities. Haller et al. [7] described apicoposterior segmentectomy with improved exercise tolerance, although variable intrathoracic obstruction persisted. Rebollo-Simarro and Alonso-Ojembarrena [8] reported planned surgery at 18 months without complication, although operative details were limited. Martínez et al. [29] presented the most complex example, combining fetoscopic laser decompression, amnioreduction of 1,400 mL, and postnatal middle and lower lobectomy on day 12 of life. Despite severe prenatal disease, the child was discharged without chronic treatment and had good respiratory function at 14 months.

Anomalies associated with vascular compression and central stenosis were associated with a greater peri-intubation burden. Lovett et al. [10] reported urgent surgery with reimplantation of the left pulmonary artery and tracheoplasty using a Y-shaped pericardial patch. Granulation tissue formation and delayed extubation until postoperative day 25 reflected the complexity of airway reconstruction. Li et al. [13], in a less severe case, described earlier recovery after left pulmonary artery reimplantation and atrial septal defect closure, with discharge on postoperative day eight and complete resolution of stridor. Collectively, these reports indicate that combined vascular and airway lesions often require multidisciplinary surgical planning and prolonged postoperative surveillance.

Isolated or associated TB showed a more variable clinical course. Sharma et al. [9] did not report specific treatment or follow-up. Ashmeade and Carver [30] focused treatment on congenital diaphragmatic hernia rather than the TB, although prolonged respiratory support was required. In Lovett et al. [10], the accessory bronchus formed part of a broader reconstructive airway problem and influenced postoperative extubation strategy.

BI and diffuse bronchomalacia were generally managed conservatively rather than by anatomical correction. Cappuccio et al. [14] reported improvement with antibiotics and chest physiotherapy. Adhikari et al. [15] used antibiotic prophylaxis guided by the presence of associated asplenia. Lee et al. [16] recommended physiotherapy and aggressive infection management without stenting. Bush [17] emphasized that recognition of left isomerism with diffuse bronchomalacia allowed withdrawal of a prior diagnosis of corticosteroid-resistant asthma and avoidance of unnecessary treatment.

Central TAg or TAt had the poorest prognosis, although outcomes varied according to anatomy and the presence of a tracheoesophageal fistula. Lopez et al. [1] attempted primary reconstruction, but a 1.5- to 2-cm gap precluded successful laryngobronchial anastomosis. van der Putten et al. [2] reported no feasible corrective treatment, with death occurring within hours of birth. Krishnamurthy et al. [31] similarly described a lethal anomaly without any functional rescue pathway. Ali et al. [3] achieved temporary stabilization for approximately 60 hours using esophageal ventilation, but no definitive correction was possible. Bhattarai et al. [5] performed cervical exploration, thoracotomy, distal esophageal ligation, and gastrostomy, although the infant died from postoperative sepsis. In contrast, Baroncini-Cornea et al. [4] described survival to 10 months after staged palliation, including pseudotracheostomy and carinal stenting.

The included case series also provided insight into medium-term outcomes. Baden et al. [12] reported generally favorable outcomes in three children with BB. One child required no airway surgery until seven years of age, another was weaned from ventilation and tracheostomy between six and seven years, and a third remained asymptomatic at four years. These findings suggest that a BB does not always require immediate correction when associated lesions are recognized and monitored carefully.

Overall, two practical messages emerged. First, central anomalies causing stenosis, airway interruption, or major reconstruction carried the greatest peri-intubation burden, including prolonged intubation, repeated bronchoscopy, risk of granulation, alternative airway strategies, or absence of any curative airway. Second, distal bronchial lesions and laterality disorders primarily emphasized the need for careful interpretation of ventilation and imaging. Technical success of intubation does not exclude abnormal air distribution, altered compliance, or misleading radiographic findings caused by the underlying anatomy. Therefore, treatment strategy extends beyond surgery alone and includes how anesthesia, resuscitation, and surgical teams plan, monitor, and reinterpret each stage of airway management (Table 2).

**Table 2:** Relationship between anomaly class, topography, clinical phenotype, and peri-intubation risk. This table summarizes the principal classes of congenital tracheobronchial anomalies identified in the review, together with their dominant anatomical topography, usual clinical presentation, and major risks during endotracheal intubation or peri-intubation airway management. Tracheal agenesis (TAg) or atresia (TAt) is characterized by the absence or discontinuity of the central airway and most commonly presents as a neonatal cannot-intubate emergency. Bronchial atresia (BAt) is typically associated with distal air trapping and may mimic selective intubation, mucus plugging, or obstructive pathology. Tracheal bronchus (TB) involves an aberrant origin of the right upper lobe (RUL) bronchus and increases the risk of inadvertent tube-related occlusion. Bridge bronchus (BB), with or without pulmonary artery sling (PAS), is characterized by abnormal bronchial branching and a pseudocarina, increasing the risk of landmark misidentification and unintended selective ventilation. Bronchial isomerism (BI) or laterality defects (LDs) distort typical bronchial anatomy and may complicate bronchoscopy, tube positioning, and interpretation of ventilation findings. Representative studies are provided for each anomaly class. n, number of included studies; cm, centimeters; mm, millimeters; PEEP, positive end-expiratory pressure; PPV, positive-pressure ventilation; T6-T7, thoracic vertebral levels 6-7; TEF, tracheoesophageal fistula.

Anomaly class	Dominant topography	Usual clinical phenotype	Major intubation risk	Representative studies
TAg/TAt (n = 6)	Absence or discontinuity of the central conduit; 5-mm subglottic stump; 1.3–1.5 cm blind pouch; gaps from 1.5–2 cm to >50 mm; variable TEF	Neonatal cannot-intubate, sometimes with paradoxical esophageal ventilation	Pure failure of central access; or, if esophageal atresia is also present, simultaneous failure of tracheal and esophageal routes	Lopez et al. [1]; van der Putten et al. [2]; Krishnamurthy et al. [31]; Ali et al. [3]; Baroncini-Cornea et al. [4]; Bhattarai et al. [5]
BAt (n = 4)	Segmental, lobar, or mainstem lesion; localization 3 cm distally or ~2 cm after the bifurcation; air trapping and mucocele	Incidental finding, infection, or pseudo-obstructive emphysema; sometimes fetal hydrops	False positives of selective intubation, mucus plugging, or iatrogenic hyperinflation under PPV/PEEP	Williams et al. [6]; Haller et al. [7]; Rebollo-Simarro & Alonso-Ojembarrena [8]; Martínez et al. [29]
TB (n = 3)	Aberrant supracarinal origin of the right upper lobe bronchus; sometimes post-orificial tracheal narrowing or distal complete rings	Recurrent infection, persistent RUL atelectasis, stridor, or respiratory distress	Iatrogenic occlusion of the aberrant bronchus by tip/cuff; selective lobar intubation; critical sizing when stenosis coexists	Sharma et al. [9]; Ashmeade & Carver [30]; Lovett et al. [10]
BB ± PAS (n = 3)	Caudal pseudocarina (often T6–T7), contralateral bronchial supply, abnormal aero-vascular relations	Stridor, infection, weaning failure, pseudo-asthma, or central compression	Carina/pseudocarina misidentification; inadvertent selective ventilation; tube depth misread	Pardo et al. [11]; Baden et al. [12]; Li et al. [13]
BI/LD (n = 4)	Bilateral hyparterial bronchi, bilateral bilobed lungs, long main bronchi; frequent associated bronchomalacia	Recurrent infection, refractory wheeze, pseudo-asthmatic pattern	Distorted bronchoscopic and selective-intubation landmarks; dynamic collapse mimicking bronchospasm	Cappuccio et al. [14]; Adhikari et al. [15]; Lee et al. [16]; Bush [17]

Discussion

This scoping review highlighted a key cross-cutting principle: the clinical severity of CTBVs in pediatric patients is determined less by rarity alone and more by the anatomical level at which airflow is interrupted, redirected, narrowed, or dynamically compromised. Lesions affecting the central airway, such as TAg or TAt, abolish normal tracheal access and create cannot-intubate emergencies associated with very high mortality. Fixed stenotic lesions, including complete tracheal rings or PAS, primarily create problems related to airway caliber, tube selection, traumatic intubation, and postoperative airway management. In contrast, more distal anomalies, such as BAt, TB, BB, and LDs, more commonly produce diagnostic and ventilatory pitfalls. Although often less dramatic at presentation, these abnormalities may be particularly hazardous because they can be mistaken for common technical problems such as endobronchial intubation, mucus plugging, bronchospasm, or incorrect tube depth.

The second key lesson was methodological. Restricting inclusion to reports of direct intubation mishaps would have excluded several anomalies highly relevant to anesthetic and critical care practice. Reports of BAt, BI, and, in some cases, BB were not always recognized during laryngoscopy or induction, yet they substantially influenced the assessment of tube depth, the interpretation of radiographic asymmetry, ventilation strategy, and bronchoscopic landmarks. Inclusion of anomalies with indirect but clinically meaningful airway relevance, therefore, strengthened the practical value of this review.

Regarding diagnosis, the included studies supported a pragmatic hierarchy of investigations. When a central airway abnormality is suspected because the vocal cords are visible, but the tube cannot be advanced, urgent endoscopy combined with rapid CT imaging should be prioritized when hemodynamic status permits. When intubation is technically successful but unilateral ventilation, unexplained hyperinflation, RUL atelectasis, or refractory wheeze persists, CT, CTA, or MDCT with airway reconstruction is useful for defining airway geometry, while bronchoscopy is valuable for assessing dynamic wall properties and the relationship between the tube tip, carina, pseudocarina, and aberrant bronchial orifices. In stable fetal or neonatal cases, targeted US and fetal MRI may aid triage and follow-up, although they do not replace detailed structural airway mapping.

The review also highlighted the need for more standardized reporting of congenital airway anomalies. Many case reports documented the anomaly itself but omitted operational details important to anesthesiologists, including the distance between an aberrant bronchial orifice and the carina, minimum diameter of a stenotic segment, tube size and type, insertion depth, confirmation method, ventilator settings, response to positive end-expiratory pressure, capnography findings, or immediate post-intubation imaging. In the present corpus, Sharma et al. [9] and Pardo et al. [11] illustrated this gap between good anatomical description and limited peri-intubation detail. By contrast, Lovett et al. [10], Baroncini-Cornea et al. [4], Bhattarai et al. [5], and Martinez et al. [29] provided more clinically actionable information.

Several practical recommendations emerged. Suspected TBVs should be documented topographically rather than by name alone. Terms such as TB or left isomerism are insufficient without specifying lesion level, laterality, and likely implications for tube depth or ventilation. Bronchoscopic confirmation should be used liberally when PAS, distal tracheal stenosis, pseudocarina, or supracarinal branching is suspected, as auscultation and standard radiography may be misleading. Ventilation strategies should be individualized, since conditions such as distal BAt, diffuse bronchomalacia, or extrinsic vascular compression may not tolerate conventional positive end-expiratory pressure or standard expiratory times.

Limitations

This review has limitations inherent to the available evidence. The corpus consisted mainly of case reports and small case series, which are subject to publication bias, descriptive heterogeneity, and inability to estimate prevalence or treatment effect. The design does not permit reliable inference regarding frequency, diagnostic performance, or causality. Some reports also lacked complete peri-intubation data, including tube diameter, insertion depth at the lips, cuff type, capnography findings, and detailed ventilation parameters. A small number of studies contained internal inconsistencies or limited follow-up, including discordant age reporting in Pardo et al. [11] and sparse outcome data in Sharma et al. [9]. Despite these limitations, the consistency of observed airway failure patterns and the anatomical detail of the included reports provide useful practical insights for pediatric airway planning.

## Conclusions

Pediatric CTBVs are not a homogeneous group of anatomical curiosities, but rather a spectrum of airway configurations associated with distinct risks during endotracheal intubation and perioperative airway management. The analyzed corpus demonstrated a clear gradient between central airway inaccessibility, typified by TAg or TAt, and more distal or laterality-related anomalies that more commonly impair interpretation of ventilation findings and endoscopic landmarks.

Several practical implications arise from this review. Identified abnormalities should be documented with precise anatomical level, laterality, and measurable landmarks whenever possible. CT, CTA, or MDCT and bronchoscopy should be considered complementary rather than competing investigations, as each provides different but clinically valuable information. The absence of a previously documented intubation mishap does not exclude substantial airway relevance. Peri-intubation planning should address not only the risk of complete airway failure, but also false-positive interpretations such as pseudocarina, distal air trapping, dynamic collapse, or iatrogenic lobar atelectasis. Overall, these findings support an explicitly anatomical, individualized, and well-documented approach to pediatric intubation in children with known or suspected tracheobronchial anomalies.
